# Abstract from a Discussion Following the Reading of a Paper upon "Soft Foil and Conservative Methods."

**Published:** 1896-08

**Authors:** W. T. Arrington


ARTICLE VI.

### ABSTRACT FROM A DISCUSSION FOLLOWING THE READING OF A PAPER UPON "SOFT FOIL AND CONSERVATIVE METHODS."

BY DR. W. T. ARRINGTON.

Proceedings of the Southern Dental Association in Atlanta *Dental Journal.*

Dr. W. T. Arrington read a paper upon "Soft Foil and Conservative Methods," describing in detail his method of filling certain typical cavities. The essential feature upon which the author laid stress was the method securing adaptation to the cavity-walls by means of Abbey's soft gold foil, and the avoidance of deep anchorages in the shape of retaining-pits or undercuts. Where contour was in certain cases demanded, or when cohesive gold could be employed to advantage, the major portion of the filling, and especially that in contact with the cervical margin and frail walls, having been guarded with soft foil, the remainder of the filling was to be completed with Watt's crystal gold, No. 2. In cases where gold seemed to be incompatible with tooth-structure or oral fluids, the author recommended for bicuspids and molars the combination of gold and tin foil, equal parts.

The treatment which the author employed for cavities with near exposure of the pulp was detailed at length, and followed well-known conservative lines in the application of plastics, either alone or in combination. The author called attention to the work in soft gold done by well-known practitioners of forty or fifty years ago, and expressed the belief that a larger percentage of their fillings are standing to-day than will be standing forty years hence of those made of cohesive gold.

DISCUSSIONS.

Dr. E. P. Beadles: Dr. Arrington spoke of his methods as being perhaps considered *old fogy*; but I agree with him in almost every point. I differ a little with him in methods, and do not always agree with him as to materials. His ideas, however, are not "old fogy" if that means obsolete, or out of date.

Dr. G. F. S. Wright: I have listened to Dr. Arrington's paper with much pleasure; but whenever members of the dental profession bring up the methods of forty years ago, they take us younger members at a disadvantage. He fails to tell us how to restore the V-shaped spaces they made; how to knuckle up and restore the true shape of the teeth, as we do with cohesive gold. I would thank him to tell us how to do that with soft foil. An anchor screw will retain such a filling made with cohesive gold as could never have been made with soft foil. The fillings of forty years ago were rammed in by hand-pressure sufficient to dislocate the jaw; in fact, it sometimes did. They are continually ringing the changes on the teeth that have "stood for forty years" filled with soft foil. I don't doubt but that it is so; but you can save a tooth for ten years with cohesive gold that you couldn't save at all with soft foil. I quite agree with all that he has said of plastics.

Dr. L. G. Noel, Nashville, Tenn: I admire the concise perspicacity of the paper, and I understand the value of plastics; but I consider it unprofitable to rake up the old fight of soft gold *versus* cohesive, Soft gold has its uses, and I do not entirely discard it, but use it in many cases now. But since the fight was first instituted there have been great improvements in cohesive gold. I am not sure that we get as good adaptation with cohesive gold as we can with soft gold.

Dr. T. M. Allen: Whenever we discuss operative dentistry, we get on to the comparative value of materials. It makes no difference what material we use if the material used is properly inserted. Dr. Arrington is right in what he says about the preparation of cavities. There is more in that than in the material we use. Anything that is so inserted as to be moisture-tight will preserve the tooth. Soft gold is properly non-cohesive gold, but it can be made cohesive by heat. I prefer to use soft gold when the cervical wall is gone, but I finish with cohesive gold.

Dr. J. Y. Crawford: I did not hear all of Dr. Arrington's paper, but was pleased with what I did hear. Many things can be done with so-called soft foil; the distinction drawn should be cohesive and non-cohesive foil. In my candid opinion, there should generally be such a handling of cohesive foil as will make it non-cohesive. Soft or non-cohesive gold, particularly that furnished by Charles Abbey, is a better tooth-preservative than any other one form of gold ever made.

Dr. W. E. Walker: In the discussion of the paper on pulp-capping, last night, some points were touched upon that will bear further discussion.

When Dr. Crawford spoke of the zone of non sensitiveness near the pulp, in a near exposure, I thought he was going to speak of two conditions which I have observed when the dentine is non sensitive. I have observed this condition of non-sensitiveness, but never the restoration of sensitiveness after twelve or twenty-four hours of which he spoke.

But I have found one or two conditions usually prevailing in this non-sensitive zone. Either the non-sensitiveness results from infiltration of calcic matter, which condition is not so unfavorable, or the contents of the tubuli will be found putrescent. In the first-named condition the dentine is hard and usually of nearly normal color; but in the second condition the color may vary from normal to dark gray, and upon cutting into the dentine it will be found softer than normal; and upon cutting down through it, we are apt to find that portion of the pulp immediately adjacent to the non-sensitive dentine in a dead and often decomposing condition, forming what might be called a pocket of pus and gases. In this condition, *hot* applications give pain, causing expansion of the gases, producing pressure upon the still vital portion of the pulp. This illustrates the importance of differential diagnosis. Dr. Jack's subdivision of the conditions of the pulp into the subjective and the objective stages is a valuable aid in diagnosis and treatment. A knowledge of temperaments is important in its bearing upon pulp-capping. I find a person of the nervous temperament a much better subject for pulp-capping than the lymphatic. They have much greater vitality and power of recuperation. They can go very near to the point of death and come up again, while a person of lymphatic temperament, having lower vitality, would succumb. It is more difficult to devitalize a pulp for a person of nervous temperament, and hence our better success in pulp capping. The habit of the patient and the general health must be considered. We must study the patient as a whole. If from sedentary occupation, careless habits, or general disregard of the laws of health,—if from these or other causes the functions of the alimentary canal are sluggish, the colon habitually clogged,—it is better not to cap pulps for that patient. We must select our cases. I have an illustration of this in the case of a man who called himself a dentist, and who "practiced" upon the public in my town for a while. He had an absolute specific for capping pulps,—capped them all indiscriminately, even capping portions of pulps; did not care whether they were inflamed or not; a suppurating pulp made no difference; his capping saved them all. I tried to reason with him, and urged him to discriminate, but he had absolute faith in his pulp-capping specific, which he even talked of patenting if he could devise any means of also keeping his secret (and which, by the way, as he subsequently told me, was iodoform, creosote and oxyphosphate). In about a year I met him, and again asked him if he was still as successful as ever in pulp-capping. Oh, no!" he replied. "I never cap pulps now." Nothing in the world can save an exposed pulp. The inevitable result of capping pulps indiscriminately must, in a discouragingly large proportion of cases, be *failure*, and this he had learned to his sorrow.

Dr. Gordon White: As a young man, Dr. Patrick discouraged me so about capping pulps that I concluded he must be right; and having lost a good many capped pulps, for a while I destroyed almost every pulp, whether it came to me exposed or whether I exposed it myself. I believed it was the correct thing to do it, invariably. But after getting teeth more exclusively under my own care, I concluded to risk capping some that were clearly not dying. I thought perhaps it might be wrong to destroy all, so I began capping pulps again. I tried every method I could hear of, but, except in rare instances, I invariably lost them. But I learned to select my cases, and now rarely try to cap a pulp that has ached. As I said in my paper, for some time I have only taken the risk in two cases, as an experiment. Twelve years ago, in 1883, I capped a pulp for Dr. Noel. It had not really ached, but had given him an uneasy sensation. When I examined the tooth, I found an exposure which bled slightly, but I capped it, and that pulp is still living, as Dr. Noel, who is here, will testify.

I did not think it necessary, in my brief paper, to go into the conditions which justify capping It is conceded that we do not attempt to save a tooth that has ached. I have inquired of my professional brethren who might possibly have fallen heirs to those that gave trouble. I have made most diligent inquiry, but I cannot find that any have given trouble. It is mistaken practice to destroy a pulp that has been accidentally exposed. I quite agree with what Dr. Walker has said in regard to temperaments, and have noted for many years that we have less success with persons of phlegmatic temperament All will agree upon that if they have given the subject any attention.

Dr. Noel: It gives me great pleasure to corroborate what Dr. White has said of the tooth he capped for me.

I wish to make one statement before the subject is dismissed. If the exposure is very extensive, we may have the misfortune to have some pressure upon the pulp from the capping; in which case, sooner or later, it will become necessary to devitalize the pulp before the tooth can be made perfectly comfortable. I am still in favor of using oxychloride of zinc and creosote, as suggested years ago. In the salvation of decidious teeth, I think I have found a place for copper amalgam. In small coronal or approximal cavities in children's teeth, when we do not need something that shall last very long, a good copper amalgam is the very best thing. Before it wastes away it will have subserved its purpose for that tooth, often, if it lasts only two years. It is decidedly antiseptic, and in children's teeth, where the cavity is small and not near the pulp, I use it all the time.

Dr. R. C. Young: But what becomes of the copper that is washed out? And what if it is carried into the alimentary canal?

Dr. Noel: There need be no uneasiness on that score. It will not poison the child.

Dr. Young: I am not so sure about that.

Dr. F. Peabody: I can testify to the value of copper amalgam in children's teeth.

I have in mind a case in which I tried indefatigably to save the teeth. I tried tin and amalgam in the approximal cavities, but they would fail in one or two months, until the teeth were nearly gone. I tried the ordinary alloys, cement, gutta percha, tin. Finally, in despair, I tried copper amalgam. That was six years ago, and there has been no further decay. It has accomplished what I had not been able to do with any other material, and I tried them all. As to the wasting of copper-alloy fillings, I can't say what becomes of it, but for the first permanent molars it has been in, I cannot percieve that it has any effect in any way except the good effects of saving the teeth, and I think it will stay there as long as it is needed.

Dr. Marshall: I cannot say that I cap all pulps that haver never ached, bled, etc. I am satisfied that Dr. White has the success he claims, but I draw the line at pulps that have been traumatically exposed, but without inflammation, pus, etc.

Dr. C. R. Young: In my experience, nine out of ten capped pulps die, though I think I exercise all possible care. I have many little children under my care, and I want to do the right thing by them. But what can you do when they come to you with the first permanent molars just through the gum, and the parents demand the extraction of abscessed aching deciduous teeth? What can you do in such cases? The roots are probably partially absorbed, and you cannot clean and fill them; you would go right through into soft tissue, or you might strike the per manent tooth germ. I can manage little children all right, but I do not know how to manage such cases.

Dr. W. E. Walker: My practice with devitalized deciduous teeth is to fill them by the method suggested by Dr. Cravens a number of years ago, though in their treatment and antiseptic medication prior to filling I differ from him. I clean out the cavity and root canals cautiously and carefully, and as thoroughly as possible consistent with due consideration for the little patient. As these teeth have but a limited time to serve, they can of course bear less preparation than in what we call permanent work. I then pump or churn in phosphate of lime, in the magna state, working into this soft mass the dry phosphate of lime, filling the cavity as usual. If this root filling goes through it does no harm, and if there is further ab sorption of the root (as sometimes occurs in replanted or implanted teeth) there will be no stiff filling projecting beyond, forming an obstacle in the path of the incoming teeth.—*Atlanta Dental Journal.*

